# Phage Infection in Vaginal Lactobacilli: An *In Vitro* Study

**DOI:** 10.1155/S1064744997000094

**Published:** 1997

**Authors:** Sylvia I. Pavlova, Ali O. Kiliç, Susan M. Mou, Lin Tao

**Affiliations:** 1 Department of Obstetrics and Gynecology School of Medicine University of Missouri-Kansas City Kansas MO USA; 2 Department of Oral Biology School of Dentistry University of Missouri-Kansas City 650 East 25th Street Kansas MO 64108 USA; 3 Department of Microbiology and Clinical Microbiology School of Medicine Karadeniz Technical University Trabzon 61080 Turkey

## Abstract

*Objective:* During bacterial vaginosis, an unexplained decrease of vaginal lactobacilli occurs. To identify whether these lactobacilli could be infected by phages, we isolated phages from vaginal lactobacilli and analyzed their potential virulence in attacking vaginal lactobacilli *in vitro*.

*Methods:* Vaginal samples were obtained from 39 reproductive-aged women. The selective Rogosa SL agar was used to isolate lactobacilli, from which phages induced by mitomycin C or released spontaneoulsy were analyzed by the agar spot method.

*Results:* Of 20 samples from women with vaginal infections, 12 did not have lactobacilli. From the remaining 8 infection samples and the 19 samples from healthy women, 37 *Lactobacillus* strains were isolated, from which 7 temperate phages were identified. Upon analysis, all 7 phages infected vaginal lactobacilli from the same and/or different women *in vitro*. Two phages, Φkc005 and Φkc007, had a broad host range, infecting 7 of 8 species tested. A control intestinal *Lactobacillus* phage also lysed several vaginal strains. One vaginal phage, Φkc039, was apparently lytic against vaginal lactobacilli from 7 other women. This phage was characterized as follows: plaque morphology, small and clear; burst size, 300 phages per cell; spontaneous induction rate, 1 per 10^6^ cells; DNA, double-stranded and linear, 41 kb; and shape, a hexogonal head and a non-contractile tail.

*Conclusions:* Bacteriophages were isolated from vaginal lactobacilli of some women and were shown *in vitro* to lyse vaginal *Lactobacillus* strains from the same and/or different women. It was suggested that vaginal lactobacilli might be suppressed by phages.


Infectious Diseases in Obstetrics and Gynecology 5:36–44 (1997)
© 1997 Wiley-Liss, Inc.

### Phage Infection in Vaginal Lactobacilli: An *In Vitro* Study

**Sylvia I. Pavlova,[1] Ali O. Kiliç,[2] Susan M. Mou,[1] and Lin Tao[1,2]***
[1]*Department of Obstetrics and Gynecology, School of Medicine, University of Missouri-Kansas City, Kansas City, MO*
[2]*Department of Oral Biology, School of Dentistry, University of Missouri-Kansas City, Kansas City, MO*

### Abstract

***Objective:*** During bacterial vaginosis, an unexplained decrease of vaginal lactobacilli occurs. To identify whether these lactobacilli could be infected by phages, we isolated phages from vaginal lactobacilli and analyzed their potential virulence in attacking vaginal lactobacilli *in vitro*.

***Methods:*** Vaginal samples were obtained from 39 reproductive-aged women. The selective Rogosa SL agar was used to isolate lactobacilli, from which phages induced by mitomycin C or released spontaneoulsy were analyzed by the agar spot method.

***Results:*** Of 20 samples from women with vaginal infections, 12 did not have lactobacilli. From the remaining 8 infection samples and the 19 samples from healthy women, 37 *Lactobacillus* strains were isolated, from which 7 temperate phages were identified. Upon analysis, all 7 phages infected vaginal lactobacilli from the same and/or different women *in vitro*. Two phages, ϕkc005 and ϕkc007, had a broad host range, infecting 7 of 8 species tested. A control intestinal *Lactobacillus* phage also lysed several vaginal strains. One vaginal phage, ϕkc039, was apparently lytic against vaginal lactobacilli from 7 other women. This phage was characterized as follows: plaque morphology, small and clear; burst size, 300 phages per cell; spontaneous induction rate, 1 per $10^6$ cells; DNA, double-stranded and linear, 41 kb; and shape, a hexogonal head and a non-contractile tail.

***Conclusions:*** Bacteriophages were isolated from vaginal lactobacilli of some women and were shown *in vitro* to lyse vaginal *Lactobacillus* strains from the same and/or different women. It was suggested that vaginal lactobacilli might be suppressed by phages. Infect. Dis. Obstet. Gynecol. 5:36–44, 1997. © 1997 Wiley-Liss, Inc.

Key words
*Lactobacillus* phage; lysogen; bacteriolysis; bacterial vaginosis

Indigenous vaginal lactobacilli play an important role in maintaining vaginal health.[1] However, an unexplained decrease of vaginal lactobacilli occurs during bacterial vaginosis (BV).[2–4] Women who suffer from BV may have an increased, milk-like discharge that has an unpleasant odor.[5] Although BV itself may be a mild disease, its sequelae include pelvic infections, premature labor, low birth weight, and mid-trimester pregnancy loss.[6] BV can thus be a serious disease state. The cause of BV is unknown, but recently BV has been defined as a condition in which the normal *Lactobacillus*-predominant vaginal flora is replaced with anaerobic bacteria, *Gardnerella vaginalis*, and *Mycoplasma*

Contract grant sponsor: Concerned Parents for AIDS Research/American Foundation for AIDS Research; Contract grant number: 02069-15-RG; Contract grant sponsor: University of Missouri Research Board; Contract grant number: K-3-40532.
Dr. Kiliç is now at the Department of Microbiology and Clinical Microbiology, School of Medicine, Karadeniz Technical University, 61080 Trabzon, Turkey.
*Correspondence to: Dr. Lin Tao, Department of Oral Biology, School of Dentistry, University of Missouri-Kansas City, 650 East 25th Street, Kansas City, MO 64108. E-mail: taol@smtpgate.umkc.edu.

Received 16 October 1996

Accepted 31 March 1997

*hominis*.[6] Several possible mechanisms by which vaginal lactobacilli decrease have been proposed. These include douching,[7] the use of spermicide such as Nonoxynol-9,[8] and treatment with antibiotics for other infections. However, it is unknown whether vaginal lactobacilli can decrease by natural causes.

Lactobacilli produce lactic acid and hydrogen peroxide ($H_2O_2$), which can prevent the overgrowth of other microorganisms in the vagina,[2–4] including *Escherichia coli*,[9,10] *G. vaginalis*,[11] *Chlamydia trachomatis*,[12] and *Neisseria gonorrhoeae*.[13] However, during BV, the number of $H_2O_2$-producing lactobacilli decreases drastically, while many anaerobic bacteria, such as *G. vaginalis, M. hominis, Ureaplasma urealyticum*, and *Mobiluncus* species, overgrow.[2–4] It is currently unknown whether vaginal lactobacilli decrease first, allowing the BV-associated anaerobes to overgrow, or the anaerobes overgrow first, suppressing vaginal lactobacilli. Because these anaerobes are sensitive to lactic acid and $H_2O_2$ produced by vaginal lactobacilli, theoretically they should not outgrow lactobacilli. Therefore, it is possible that lactobacilli decrease first in the vagina before the anaerobes overgrow. If so, other yet unidentified factors might inhibit vaginal lactobacilli.

A natural inhibitor of bacteria is bacteriophage or phage, the virus that infects bacteria.[14] Phages can exist as free viruses in the environment or as parasites in the host bacteria. A bacterium that hosts a latent phage (prophage) is called lysogen, which can release a small number of phages spontaneously or a large number of phages upon induction. Phages can be lytic (virulent) or temperate. The lytic phages can lyse bacteria completely, releasing many new phages into the environment. The temperate phages do not lyse all the infected bacterial cells; they convert some of them into lysogens and coexist with them. However, some temperate phages may become lytic due to mutations of the phage or changes of the bacterial host background.[14,15]

*Lactobacillus* phages have been isolated from various sources, including dairy products,[16] sausage,[17] human intestines,[18] and sewage.[19] To date, studies on *Lactobacillus* phages have mainly focused on the food fermentation industry that uses lactobacilli as starter cultures.[16–18] These foods include yogurts and fermented meats, such as sausages. A study of the production of salami dry sausage showed that phage infection can delay acid production by the *lactobacillus* starter culture and drastically reduce the number of lactobacilli in the sausage and disrupt the ripening process of the sausage.[17] Likewise, phages may also infect vaginal lactobacilli, delay their acid production, and reduce their numbers. However, whether phages can infect human vaginal lactobacilli has not been reported. Therefore, the purposes of this study were to isolate phages from vaginal lactobacilli, to determine their infectivity and virulence by cross-infecting these phages against a collection of vaginal *Lactobacillus* strains, and to characterize one of the vaginal *Lactobacillus* phages.

### SUBJECTS AND METHODS

### Isolation of Vaginal Lactobacilli

The clinical research was approved by the Truman Medical Center Institutional Review Board in Kansas City, MO. Vaginal samples were obtained from 39 reproductive-aged, non-pregnant women. All subjects gave written informed consent. First, a sample was obtained to diagnose BV. As previously described,[5] the diagnosis of BV was based upon the presence of a vaginal discharge with three of the four following characteristics: a homogenous appearance; a pH > 4.5; the presence of a fishy amine odor upon the addition of 10% potassium hydroxide (KOH); and the presence of clue cells. The vaginal discharge of healthy women had no more than one of these four characteristics, and none contained clue cells. Then, a vaginal sample was taken for isolating lactobacilli. A BBL Culturette Collection and Transport System (Becton Dickinson Microbiological Systems, Cockeysville, MD) was used. The Culturette tube contains a rayon swab attached to a cap by a plastic stick and an ampule with 0.5 ml of modified Stuart's transport medium. A swab was inserted into the vagina, rotated a few turns along the vaginal sidewall, and allowed to absorb for a few seconds before withdrawing. The swab was then placed back into the tube containing the transport medium and sent to the laboratory for analysis. The samples were inoculated onto the selective Rogosa SL agar plates (Difco, Detroit, MI) for isolating vaginal lactobacilli. The agar plates were incubated in a candle jar at 37°C for 48 h. Lactobacilli were identified on the basis of growth on the selective Rogosa SL agar

(pH 5.2), rod cell morphology, positive Gram stain, and negative catalase reaction. Further identification of the species of these lactobacilli was based on carbohydrate fermentation patterns and gas production in the lactobacilli *MRS* broth (Difco, Detroit, MI) described in *Bergey's Manual of Systematic Bacteriology*.[20] Purifed cultures were maintained at –70°C in MRS broth with 10% glycerol. Two vaginal *Lactobacillus* type strains, *L. gasseri* ATCC 9857 and *L. jensenii* ATCC 25258, were obtained from the American Type Culture Collection (Rockville, MD) to be used as controls. Additional control strains included a human intestinal strain, *L. gasseri* ADH and its phage ϕadh (from Dr. Klaenhammer, University of North Carolina), and a dairy type strain, *L. delbrueckii* subsp. *lactis* ATCC 15808.

### Phage Induction

Mitomycin C (Sigma, St. Louis, MO) was used to induce phages from vaginal lactobacilli as previously described.[16] The induction of *Lactobacillus* prophages was indicated by the lysis of a *Lactobacillus* culture 4–7 h after the addition of mitomycin C. These lysates were then centrifuged, filtered through a 0.2 μm pore-size filter, and maintained at 4°C with a drop of chloroform.

### Phage Infectivity Assay

Phage infectivity was determined by the agar spot method as previously described.[16] All positive results were verified by single plaque formation, phage DNA isolation, and observation of phages under an electron microscope to rule out possible bacterial inhibitory effects due to bacteriocin, $H_2O_2$, or organic acids.

### Phage Titration

The temperate (non-virulent or non-lytic) phage, ϕkc039, from a vaginal *Lactobacillus* isolate (*L. delbrueckii* subsp. *delbrueckii* KC039), was found to be lytic (virulent) against another vaginal isolate, *L. delbrueckii* subsp. *delbrueckii* KC013. Thus, KC013 was used as an indicator strain to analyze the titer of ϕkc039. Plaques were counted after 24 h of incubation at 37°C on MRS agar with 10 mM $CaCl_2$.

### One-Step Growth Curve

KC013 was grown at 37°C in MRS broth until mid-exponential phase. To 1 ml of this culture, 0.1 ml of ϕkc039 stock [$10^8$ plaque forming unit (PFU)/ml; multiplicity of infection, 1:10] was added and mixed. The mixture of cells was added into 4 ml of melted soft 0.6% MRS agar with 10 mM $CaCl_2$, rapidly mixed, poured onto MRS plates, and incubated at 37°C. Plaques were numerated after 24 h of incubation.

### Spontaneous Phage Induction

KC039 was grown in 2 ml of MRS broth to mid-exponential phase. One milliliter of the culture was diluted and plated on MRS agar plates for cell count. Another 1 ml was centrifuged to harvest the supernatant, which was filtered through a sterile 0.2 μm pore-size filter. The supernatant was diluted and used to infect the indicator strain KC013 by the soft agar overlay method as described above. Plaques were numerated after 24 h of incubation at 37°C.

### Phage DNA Analysis

The bacteriophage ϕkc039 was purifed from 1 l of mitomycin-induced lysate by a standard method.[21] The phage DNA was extracted with the QIAGEN (Chatsworth, CA) lambda phage DNA isolation kit. Restriction enzyme digests of ϕkc039 DNA were subjected to gel electrophoresis on a 0.8% agarose gel at 40 V for 3 h. The gel was stained with ethidium bromide and photographed under an ultraviolet light.

### Electron Microscopy

One drop of the purifed phage ϕkc039 in 0.1 M ammonium acetate (pH 7.0) was spotted on grids with a carbon-coated Formvar film (Ladd Research Industry, Burlington, VT). After drying for 30 sec, the sample was negatively stained with 2% uranyl acetate (pH 4.2). Electron microscopy was performed with the CM12 transmission electron microscope (Philips Electronic Instruments, Inc., Mahwah, NJ) at 80 kV.

### RESULTS

### Isolation and Identification of Vaginal Lactobacilli

Vaginal samples were obtained from 39 reproductive-aged women (1 Native American, 5 Asians, 6 Blacks, and 27 Caucasians; age range: 19–52 years). Evaluation of the vaginal secretions revealed 19 normal women, 16 women with BV, and 4 women with vaginal candidiasis (VC). All women with the clinical diagnosis of BV, the presence of at least three of four Amsel's criteria,[5] had a reduction or absence in vaginal lactobacilli. VC was confirmed by the growth of *Candida* cells in the culture test. Lactobacilli were isolated from all of the 19 healthy women and 4 VC patients, but from only 4 of 16 women who had BV. Some vaginal samples had multiple *Lactobacillus* strains/species; therefore, a total of 37 *Lactobacillus* strains were isolated from the 27 women. Based on sugar fermentation patterns, the species of the 37 vaginal *Lactobacillus* isolates were tentatively identified as follows: *L. acidophilus,* 10 strains; *L. delbrueckii* subsp. *delbrueckii,* 19 strains; *L. fermentum,* 3 strains; *L. jensenii,* 4 strains; and *L. plantarum,* 1 strain.

### Phage Isolation and Cross-Infection

Phage induction was performed with the mitomycin C method in 37 clinically isolated vaginal strains, 2 control vaginal type strains (*L. gasseri* ATCC 9857 and *L. jensenii* ATCC 25258), and 2 other control strains (*L. gasseri* ADH and *L. delbrueckii* subsp. *lactis* ATCC 15808). The lysates were used to interact with the 41 *Lactobacillus* strains to screen for phage-sensitive indicator strains. Seven lysates were confirmed to contain phages because they formed single plaques on the agar plates of sensitive strains. Among the 7 phages, 3 were from 29 *Lactobacillus* strains of 19 healthy women, 2 from 4 strains of 4 VC patients, and 2 from 4 strains of 4 BV patients. Although the rate of phage isolation was apparently lower from lactobacilli in healthy women than in women with BV or VC, there was no apparent difference in phage sensitivity among vaginal *Lactobacillus* strains isolated from these women. The sources and host ranges of these phages and the sensitivity of various vaginal *Lactobacillus* isolates to these phages are shown in Table 1. Strains from all *Lactobacillus* species studied, except *L. fermentum,* were infected by phages. The phage ϕkc005 isolated from a vaginal *L. acidophilus* strain of a women with VC had a broad host range and was very virulent. Unlike other phages, ϕkc005 could continue to spread its infection on a soft agar plate by enlarging its lytic zones with time (Fig. 1). Interestingly, the control phage isolated from the intestinal strain *L. gasseri* ADH also lysed 6 of these vaginal strains.

### *In Vitro* Study of Vaginal *Lactobacillus* Phage Infection

All 7 temperate phages isolated from vaginal lactobacilli lysed vaginal lactobacilli from the same and/or different women *in vitro* (Table 1). Among these phages, the temperate phage, ϕkc039, isolated from a vaginal *Lactobacillus* strain, KC039, was found to be lytic to 7 other vaginal *Lactobacillus* clinical strains and 1 vaginal type strain. Among the 8 strains, KC013 was the most sensitive one to ϕkc039 infection. A bacterial strain that is sensitive to a phage by showing plaques on agar plate upon infection can be used to analyze the phage as an indicator strain. Thus, KC013 was selected as the indicator strain for analyzing ϕkc039. Normally, temperate phages only partially lyse their bacterial indicator strains by showing turbid plaques on agar plates, such as the case of ϕkc005 (Fig. 1). However, ϕkc039 showed clear plaques after the phage infected *Lactobacillus* strain KC013 on agar plates (data not shown). The complete lysis of KC013 by ϕkc039 allowed isolation of a large amount of phage particles and genomic DNA. This greatly facilitated characterizations of this phage. Moreover, the phage ϕkc039 and its indicator strain KC013 formed an ideal *in vitro* model to show that a temperate phage released from a vaginal *Lactobacillus* stain of one woman could become a lytic phage against a different vaginal *Lactobacillus* strain from another woman.

### Characterization of ϕkc039

The infection cycle of ϕkc039 against its new host strain KC013 in MRS broth with 10 mM $CaCl_2$ was characterized by its one-step growth kinetics. Figure 2 graphically represents the increase of PFU/ml as a function of time during phage infection. The events in bacteriophage development were determined: the time between adsorption and lysis (latent period) was 30 min; the rise period was 90 min; and the average number of phage particles released per infected host cell (burst size) was about 300 phages per cell. The plaque morphology of ϕkc039 was small and clear, with a diameter ranging from 0.5 to 1 mm. The spontaneous induction rate of KC039 was about 1 in $10^6$ cells. The restriction pattern of ϕkc039 DNA is shown in Figure 3. The genome size of ϕkc039 was estimated to be 41 kb

**TABLE 1. Sensitivity of vaginal lactobacilli to phages released from different lactobacilli**

| | | Vaginal *Lactobacillus* phages | | | | | | | Intestinal phage: *L. gasseri* |
|---|---|---|---|---|---|---|---|---|---|
| | | *L. acidophilus* | | | | *L. delbrueckii*[d] | | | |
| *Lactobacillus* strain[a] (N = 41) | | ϕkc005[b] | ϕkc007 | ϕkc012[b] | ϕkc021[c] | ϕkc023[c] | ϕkc031 | ϕkc039 | ϕadh |
| Vaginal isolates (N = 37) | | | | | | | | | |
| *L. acidophilus* | KC001a | + | + | − | + | − | − | − | + |
| | KC007a | + | + | − | − | − | − | − | − |
| | KC012[b] | + | + | + | − | − | − | − | − |
| | KC021[c] | + | + | − | − | − | − | − | − |
| | KC026[c] | + | + | − | − | − | + | + | − |
| *L. delbrueckii*[d] | KC003 | + | + | − | − | − | − | − | − |
| | KC004a | + | + | − | − | − | − | + | − |
| | KC004b | + | + | − | − | − | + | + | − |
| | KC006a | + | + | − | + | − | − | − | + |
| | KC006b | + | + | − | − | − | − | − | − |
| | KC010 | + | + | − | − | − | + | − | − |
| | KC013 | + | + | − | + | + | − | + | − |
| | KC023[c] | + | + | − | − | − | + | + | − |
| | KC027[c] | + | + | + | − | − | − | − | − |
| | KC029 | + | + | + | − | − | + | + | − |
| | KC031 | + | + | − | − | − | − | + | + |
| | KC032 | + | + | − | − | − | − | − | + |
| | KC033 | + | + | − | − | − | − | − | − |
| | KC034 | + | + | − | + | − | + | − | + |
| | KC036b | + | + | − | + | − | + | − | + |
| | KC038 | + | + | − | − | − | − | − | − |
| | KC039 | + | + | − | − | − | − | − | − |
| *L. jensenii* | KC008[b] | + | + | + | − | − | − | − | − |
| | KC009 | + | − | + | − | − | − | − | − |
| | KC035 | + | + | + | − | − | − | − | + |
| *L. plantarum* | KC020 | + | + | + | − | − | − | − | − |
| Control strains (N = 4) | | | | | | | | | |
| *L. gasseri* | ATCC 9857 | + | + | + | + | − | − | − | + |
| *L. jensenii* | ATCC 25258 | + | + | − | + | − | − | + | + |
| *L. lactis*[d] | ATCC 15808 | + | + | + | + | − | − | − | + |

[a]Only phage-sensitive strains are shown; 11 vaginal strains and 1 control strain, ADH, were not infected by these phages. To multiple strains isolated from the same woman, a lowercase letter is added to their strain designations.
[b]Isolated from women with VC.
[c]Isolated from women with BV.
[d]Subspecies of *L. delbrueckii*.

by an average of the sums of the molecular sizes of these restriction fragments. The agarose gel electrophoresis, which allows discrimination among covalently closed supercoiled circular, nick-relaxed circular, and linear DNA molecules showed only one distinct band of undigested phage ϕkc039 DNA (data not shown). This indicated that the phage genome was a double-stranded linear DNA. The electron micrograph illustrates that the phage ϕkc039 has a hexagonal head, about 67 nm in diameter, and a flexible but non-contractile tail about 250 nm in length with about 60 discs (Fig. 4). This phage morphology apparently belongs to Bradley type B phages.[22]

### DISCUSSION

BV is a common vaginal infection that may result in serious sequelae, such as increased risk of preterm delivery, low birth weight, and mid-trimester loss.[6] Although the exact cause of BV is unknown, it has been well documented that during BV, the normal, *Lactobacillus*-predominant vaginal flora is replaced with anaerobic bacteria.[2–6,23] However, because vaginal anaerobic bacteria are normally suppressed by lactic acid and $H_2O_2$ produced by lactobacilli,[2–4] theoretically the anaerobes cannot remove lactobacilli unless the latter diminishes or disappears first.

Phages are natural inhibitors of bacteria. If

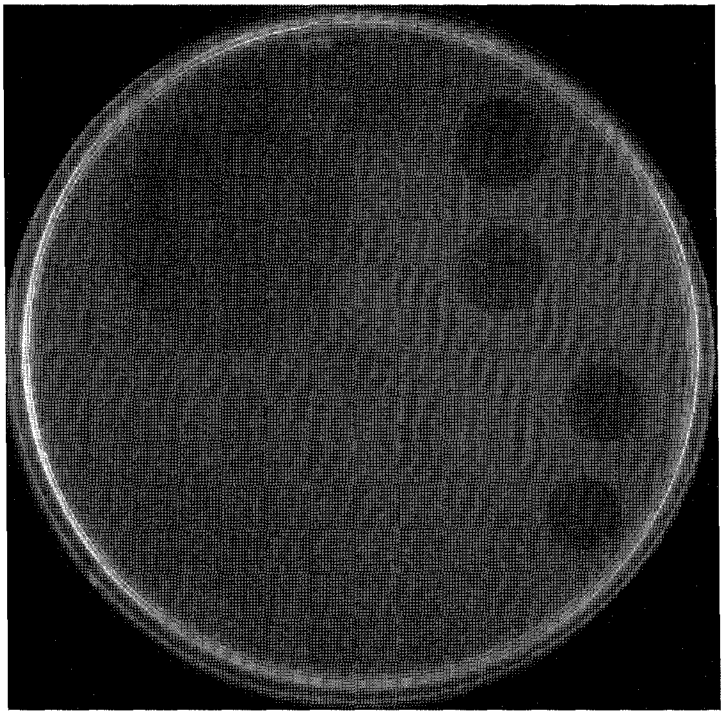

Fig. 1. *Lactobacillus* phage infections on a soft agar plate. The larger circle of bacteriolysis on the left was cause by a drop of 1 μl of ϕkc005. The four smaller circles of bacteriolysis on the right were caused by drops of 1 μl each of ϕkc012. The indicator strain was *L. delbrueckii* subsp. *lactis* ATCC 15808. Normally, the lysis of *Lactobacillus* cells would stop after 2 days of phage infection, with a lysis zone similar to the four smaller zones caused by ϕkc012. However, the infection of ϕkc005 continued as shown by the enlargement of the lytic zone with time. The result on this plate was 7 days after infection.

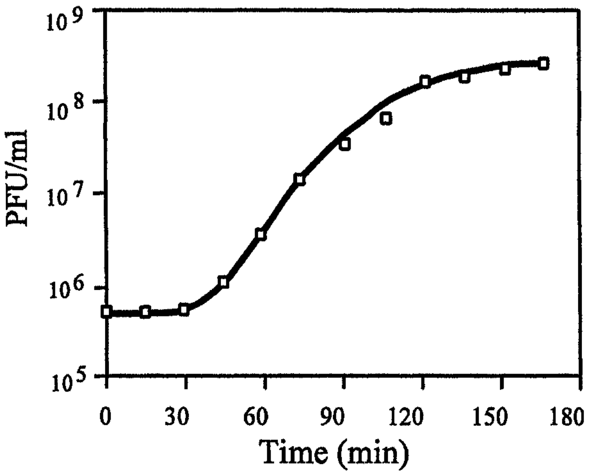

Fig. 2. One-step growth curve of vaginal *Lactobacillus* phage ϕkc039.

phages can infect vaginal lactobacilli, they may be a natural cause for the reduction of vaginal lactobacilli. Although phages have been isolated from lactobacilli of various sources,[15–19] phage infections in vaginal lactobacilli have not been reported. This study observed that some vaginal lactobacilli were lysogens that released phages spontaneously or upon induction. The rate of phage isolation from vaginal lactobacilli was apparently lower in healthy women than in women with BV or VC. As to phage sensitivity among various *Lactobacillus* species tested, *L. fermentum* did not release any phages, nor were they sensitive to phages released by other *Lactobacillus* species. Moreover, the phage ϕadh released from a human intestinal *L. gasseri* strain ADH also lysed 6 of 37 vaginal *Lactobacillus* strains tested, suggesting that infectious phages in the vagina may come from the fecal-urogenital route. However, an increased number of cases will be needed to confirm these observations.

Since it is difficult to test phage infections in vaginal lactobacilli *in vivo* in humans, *in vitro* studies were performed to show that phages released from some women's vaginal lactobacilli could lyse vaginal lactobacilli isolated from the same and/or different women (Table 1). This observation suggested that vaginal lactobacilli could be suppressed by phages *in vivo* in humans.

The analysis of these phages revealed some un-

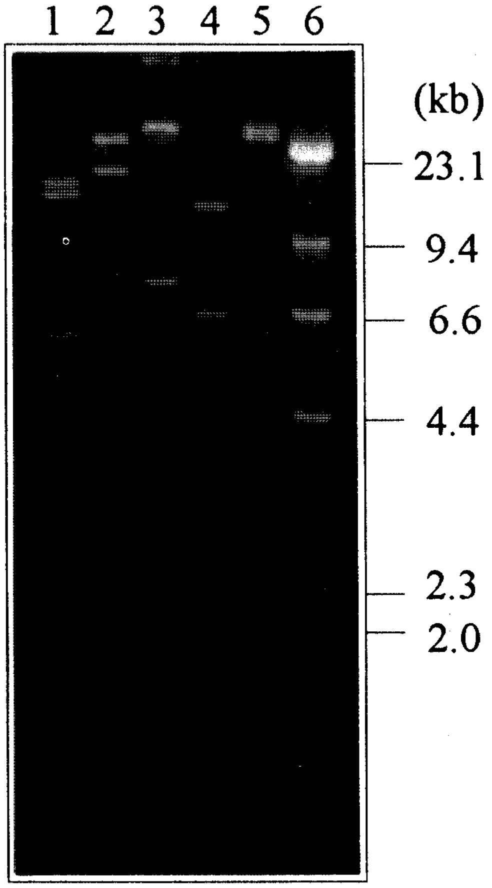

Fig. 3. Restriction analysis of vaginal *Lactobacillus* phage φkc039 DNA. Bacteriophage φkc039 DNA was digested with *Ava*I **(lane 1)**, *Bam*HI **(Lane 2)**, *Bgl*II **(lane 3)**, *Eco*RI **(lane 4)**, and *Sal*I **(lane 5)**. A *Hin*dIII digest of lambda DNA was used as the molecular weight standard **(lane 6)**.

usual and interesting characteristics. First, most phages have a specific or narrow host range, and their infection process stops when the host bacteria are not actively growing.[14,15] However, two of the vaginal *Lactobacillus* phages, φkc005 and φkc007, had a very broad host range, infecting 7 of 8 *Lactobacillus* species tested (Table 1). The phage φkc005 could also continue its infection after the host cell had stopped active growth (Fig. 1).

Second, a bacterium that carries a prophage, namely a lysogen, is normally immune from infection by other phages of the same type. This characteristic is called "super-infection immunity," which is controlled by a phage repressor protein encoded by the prophage genes already in the host bacterial chromosome or by other mechanisms.[14] This may explain why the phages released by a vaginal *Lactobacillus* strain normally would not lyse

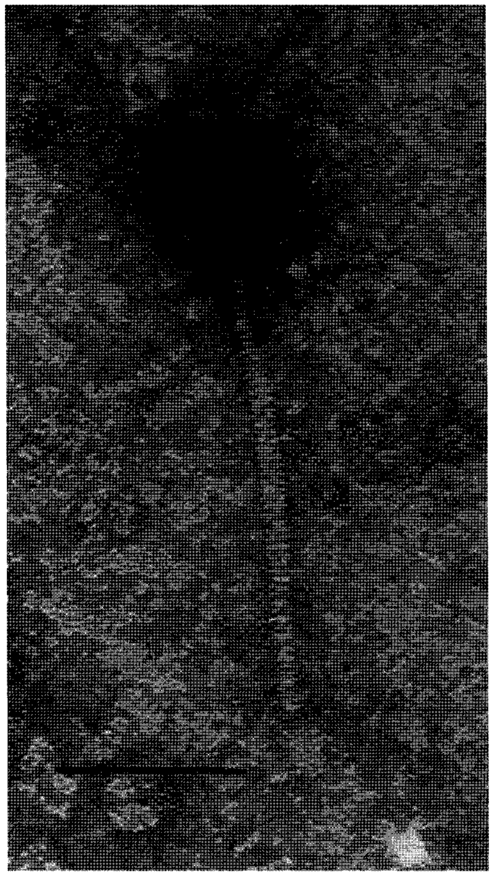

Fig. 4. Electron micrograph of the vaginal *Lactobacillus* phage φkc039. Stained with 2% uranyl acetate. ×240,000. Bar = 1,000 A (100 nm).

its own bacterial host. However, the "super-infection immunity" may not protect vaginal lactobacilli from infections by different types of phages. The observation that phages released from one *Lactobacillus* strain can lyse other lysogenic strains suggests that different types of phages may exist in these vaginal lactobacilli. But exceptions exist. Two phages, φkc007 and φkc012, lysed their own host strains, suggesting that these phages might be defective in their super-infection immunity.

Third, truly lytic or virulent phages are usually short-lived. Once they appear, the virulent phages can rapidly eliminate their host bacteria; as a result, they lose their shelter that enables them to live and to reproduce themselves. Therefore, phages that are temperate to some bacteria but lytic to others are of concern. All 7 vaginal *Lactobacillus* phages isolated in this study were temperate phages from lysogenic vaginal *Lactobacillus* strains. It is well known that some temperate phages can become virulent due to genetic mutations.[15] However, it is unknown why some temperate phages, such as ϕkc039, can become lytic against other vaginal *Lactobacillus* strains. Probably certain differences in bacterial host backgrounds prohibited these phages from integrating their DNA into the chromosome of their new hosts to form lysogens.[24] Among many characteristics of these phages, the spontaneous release of a small number of phages by lysogenic lactobacilli is noteworthy. This suggests that lysogenic vaginal lactobacilli may be a source of potentially infectious phages.

In summary, phages were isolated from vaginal lactobacilli. *In vitro* studies showed that phages isolated from vaginal lactobacilli of some women could infect vaginal lactobacilli of the same and/or different women. Also, a phage isolated from a human intestinal *Lactobacillus* strain lysed some of these vaginal lactobacilli, suggesting that infectious phages in the vagina may come from the fecal-urogenital route. Although the phage infection in vaginal lactobacilli observed *in vitro* may not necessarily represent that a similar situation could happen *in vivo*, the results imply that vaginal lactobacilli may be inhibited by phages. This implication may be important for studying the etiology of BV that is associated with the decrease of vaginal lactobacilli. However, further studies with an increased number of clinical cases will be needed to associate phage infections in vaginal lactobacilli with women's vaginal health status.

### ACKNOWLEDGMENTS

We thank Dr. T. Klaenhammer for sending us *L. gasseri* ADH strain and phage ϕadh, Dr. C. Lee, S. Robinson, and D. Sackuvich for assisting with electron microscopy, D. Coats for photography, and P. Puntenney for editing the manuscript.

### REFERENCES

1. Redondo-Lopez V, Cook RL, Sobel JD: Emerging role of lactobacilli in the control and maintenance of the vaginal bacterial microflora. Rev Infect Dis 12:856–872, 1990.
2. Eschenbach DA, Davick RR, Williams B, et al.: Prevalence of hydrogen peroxide-producing *Lactobacillus* species in normal women and women with bacterial vaginosis. J Clin Microbiol 27:251–256, 1989.
3. Hillier SL, Krohn MA, Klebanoff SJ, Eschenbach DA: The relationship of hydrogen peroxide-producing lactobacilli to bacterial vaginosis and genital microflora in pregnant women. Obstet Gynecol 79:369–373, 1992.
4. Klebanoff SJ, Hillier SL, Eschenbach DA, Waltersdorph AM: Control of the microbial flora of the vagina by $H_2O_2$-generating lactobacilli. J Infect Dis 164: 94–100, 1991.
5. Amsel R, Totten PA, Spiegel CA, Chen KCS, Eschenbach D, Holms KK: Nonspecific vaginitis: Diagnostic criteria and microbial and epidemiological association. Am J Med 74:14–22, 1983.
6. Hillier SL, Nugent RP, Eschenbach DA, Krohn MA, Gibbs RS, Martin HD, Cotch MF, Edelman R, Pastorek JG, Rao AV, McNellis D, Regan JA, Carey JC, Klebanoff MA, et al., and Vaginal Infections and Prematurity Study Group: Association between bacterial vaginosis and preterm delivery of a low-birth-weight infant. N Engl J Med 333:1737–1742, 1995.
7. Harwood B, Mittendorf R, Judge D, Dayal S, Walker C: Patterns of vaginal douching and their association with vaginal bacteriosis. Infect Dis Obstet Gynecol 4:51, 1996.
8. Hooton TM, Fennell CI, Clark AM, Stamm WE: Nonoxynol-9: Differential antibacterial activity and enhancement of bacterial adherence to vaginal epithelial cells .J Infect Dis 164:1216–1219, 1991.
9. Herthelius M, Gorbach SL, Molby R, Nord CE, Petterson L, Winberg J: Elimination of vaginal colonization with *Escherichia coli* by administration of indigenous flora. Infect Immun 57:2447–2451, 1989.
10. McGroarty JA, Reid G: Detection of a *Lactobacillus* substance that inhibits *Escherichia coli.* Can J Microbiol 34: 974–978, 1988.
11. Hallen A, Jarstrand C, Pahlson C: Treatment of bacterial vaginosis with lactobacilli. Sex Transm Dis 19:146–148, 1992.
12. Martius J, Krohn MA, Hillier SL, Stamm WE, Holmes KK, Eschenbach DA: Relationship of vaginal *Lactobacillus* species, cervical *Chlamydia trachomatis,* and vaginosis to preterm birth. Obstet Gynecol 71:89–95, 1988.
13. Zheng H, Alcorn TM, Cohen MS: Effects of $H_2O_2$-producing lactobacilli on *Neisseria gonorrhoeae* growth and catalase activity. J Infect Dis 170:1209–1215, 1994.
14. Joklik WK, Willett HP, Amos DB: Zinsser Microbiology. 17th ed. New York: Appleton-Century-Crofts, p 1178, 1980.
15. Sechaud L, Cluzel PJ, Pousseau M, Baumgartner A, Accolas JP: Bacteriophages of lactobacilli. Biochimie 70: 401–410, 1988.
16. Kiliç AO, Pavlova SI, Ma W, Tao L: Analysis of *Lactobacillus* phages and bacteriocins in American dairy products and characterization of a phage isolated from yogurt. Appl Environ Microbiol 62:2111–2116, 1996.
17. Nes IF, Sorheim O: Effect of infection of a bacteriophage in a starter culture during the production of salami dry sausage: A model study. J Food Sci 49:337–340, 1984.
18. Raya RR, Kleeman EG, Luchansky JB, Klaenhammer

TR: Characterization of the temperate bacteriophage ϕadh and plasmid transduction in *Lactobacillus acidophilus* ADH. Appl Environ Microbiol 55:2206–2213, 1989.

19. Kopeloff N: Dissociation and filtration of *Lactobacillus acidophilus*. J Infect Dis 55:368–372, 1934.
20. Kandler O, Weiss N: Genus *Lactobacillus*. In Sneath P (ed): Bergey's Manual of Systematic Bacteriology. Vol 2. Baltimore: William & Wilkins, pp 1209–1234, 1986.
21. Maniatis T, Fritsch EF, Sambrook JJ: Molecular Cloning Laboratory Manual. Cold Spring, NY: Cold Spring Harbor Laboratory Press, 1982.
22. Bradley DE: Ultrastructure of bacteriophage and bacteriocin. Bacteriol Rev 31:230–314, 1967.
23. Hill GB: The microbiology of bacterial vaginosis. Am J Obstet Gynecol 169:450–454, 1993.
24. Cluzel PJ, Serio J, Accolas JP: Interaction of *Lactobacillus bulgaricus* temperate bacteriophage 0448 with host strains. Appl Environ Microbiol 53:1850–1854, 1987.
